# High-quality genome of *Diaphanosoma dubium* provides insights into molecular basis of its broad ecological adaptation

**DOI:** 10.1016/j.isci.2023.106006

**Published:** 2023-01-18

**Authors:** Meng Xu, Ping Liu, Qi Huang, Shaolin Xu, Henri J. Dumont, Bo-Ping Han

**Affiliations:** 1 Department of Ecology and Institute of Hydrobiology, Jinan University, Guangzhou 510632, China; 2 College of Environmental Science and Engineering, Yangzhou University, Yangzhou 225127, China; 3 Ghent University, Department of Biology, Ledeganckstraat 35, 9000 Ghent, Belgium

**Keywords:** Biological sciences, Evolutionary biology, Genomics

## Abstract

*Diaphanosoma dubium* Manuilova, 1964, is a widespread planktonic water flea in Asian freshwater. Although sharing similar ecological roles with species of *Daphnia*, studies on *D*. *dubium* and its congeners are still few and lacking a genome for the further studies. Here, we assembled a high quality and chromosome level genome of *D. dubium* by combining long reads sequencing and Hi-C technologies. The total length of assembled genome was 101.8 Mb, with 98.92 Mb (97.2%) anchored into 22 chromosomes. Through comparative genomic analysis, we found the genes, involved in anti-ROS, detoxification, protein digestion, germ cells regulation and protection, underwent expansion in *D. dubium*. These genes and their expansion helpfully explain its widespread geographical distribution and dominance in eutrophic waters. This study provides insight into the adaptive evolution of *D. dubium* at genomic perspectives, and the present high quality genomic resource will be a footstone for future omics studies of the species and its congeners.

## Highlights

• Providing a high quality genome assembly of *Diaphanosoma dubium* Manuilova, 1964
• Comparative genomic analysis with published water flea genomes
• Anti-ROS genes are associated with reproductive and endozoochory adaptability
• More *CYP* and cathepsin L-like genes support high tolerance to eutrophic water

## Graphical abstract

**Figure:**
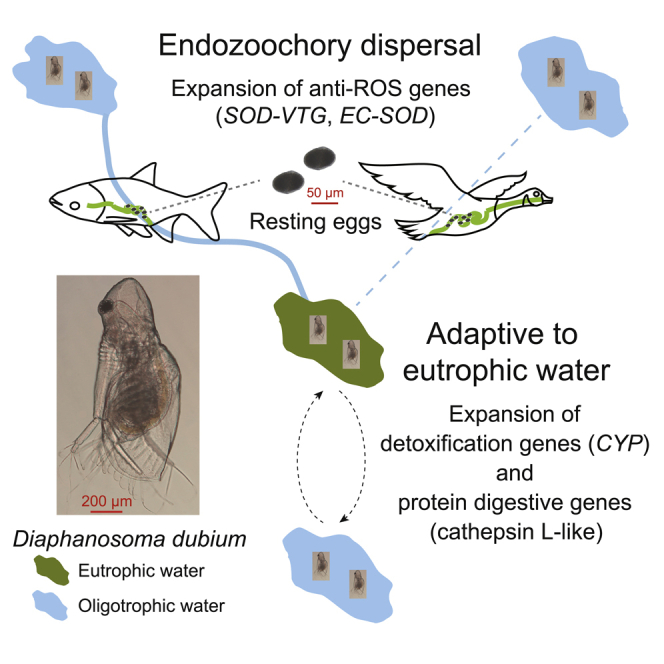

## Introduction

Cladocerans (water fleas) are keystone planktonic invertebrates and play a critical role in freshwater ecosystems by filter-feeding phytoplankton and transferring energy to higher trophic levels. They are important model or model-like organisms in ecology and ecotoxicology, as well as environmental monitoring research.^1^^,^^2^ Genetic analysis is one classical but powerful approach to explore the molecular mechanisms underlying their adaptation to diverse hostile environments. For example, whole genome sequencing for the resting eggs of *Daphnia magna* from three time periods (three depths of sediment) that varied in the level of fish-predation pressure, revealed rapid evolution of hundreds of genes in response to strong selection pressures.^3^ However, the genomes of water fleas that have been reported mainly pertain to temperate species in *Daphnia*,^4^^,^^5^^,^^6^^,^^7^ except one marine species of *Diaphanosoma*.^8^ These published genomic data promoted the studies and understanding of molecular mechanisms through omics technologies, such as comparative genomics,^9^ providing reference sequences for genome re-sequencing^3^ and transcriptome sequencing.^10^ The lack of reference genomes of water fleas in other groups, especially for (sub)tropical freshwater species, limits our understanding of the whole group of the Cladocera, as well as the further molecular studies.

*Diaphanosoma* is the largest genus of the family Sididae in the order Ctenopoda. It contains 40+ species, and occurs worldwide except in Antarctica and New Zealand,^11^ and is called “tropical *Daphnia*”. *D. dubium* Manuilova, 1964, is one of the most widespread *Diaphanosoma* species which occupies nearly the whole of Asia. It has the optimum temperature around 33°C, can grow relatively well at 38°C,^12^^,^^13^ and could be found in the near equatorial zone (Malaysia, Sri Lanka, and Philippines, etc.) and its north range could reach the Russian Far East.^14^^,^^15^ The habitats divided by sea, such as Japan and Philippines away from Asiatic mainland, suggests the existence of bird-mediated dispersal which had been reported in many aquatic organisms.^16^^,^^17^ In addition, investigations in temperate (Korea), subtropics (south China), and tropics (Philippines) all found that *D. dubium* is one of the dominant Cladocera species in eutrophic waters or during the cyanobacterial blooming seasons.^18^^,^^19^^,^^20^ With climate warming and aquatic eutrophication in the global scale, the long-distance dispersal ability and broader thermal performance^12^^,^^13^ make *D. dubium* has the potential to expand its distribution range. Actually, it has been reported the existence in its non-native regions, such as Ukraine^21^ and Ethiopian.^22^ Therefore, it needs to pay attention to *D. dubium* as an environmental indicator.

To better understand the biology, ecology, and evolution of *D. dubium*, we sequenced its genome using two technologies and assembled a high-quality genome. Considering that cyanobacteria are usually the dominant algae in eutrophic waters,^23^ and their cyanotoxins,^24^ and biochemical components are different entirely from eukaryotic algae,^25^ we identified and compared the detoxification genes (such as Cytochrome P450)^26^ and digestive enzyme genes. Furthermore, through the comparative genomic analysis, we identified the common and/or specific evolution in *D. dubium* and/or three other water fleas (*Diaphanosoma celebensis*, *Daphnia pulex*, and *Daphnia magna*), to explored the potential molecular mechanisms underlying the common or different adaptability of *D. dubium* and three water fleas. The exposed genome also provides a useful genetic resource for future studies of comparative genomics, population genetics, and other omics.

## Results and discussion

### Genome assembly and annotation

**Figure 1:**
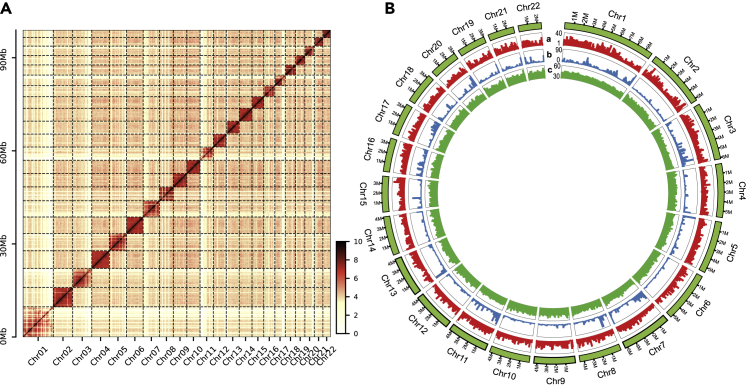
The genome assembly of *D. dubium* (A) The genome-wide Hi-C heatmap of the assembly. (B) The genomic characteristics of *D. dubium* with siding windows of 100 kb. Track a, gene density, coordinate span, 1–40; Track b, repeat content, coordinate span, 0–90; Track c, GC content, coordinate span, 30–60.

The genome of *D. dubium* was sequenced using a strategy including long reads sequencing technology (PacBio RSII platform, PB) and high-throughput chromosome conformation capture (Hi-C) technology. In all, 17.52 Gb long reads were generated with average length of 10.59 kb (Table S1). Through assembling and polishing, we achieved an assembly with total length of 101.77 Mb and Contig N50 of 1.30 Mb (Table 1). Then the contigs were clustered and assembled into pseudochromosomes with the aid of 22.4 Gb Hi-C data. As a result, 98.92 Mb (97.1%) of contigs were anchored into 22 chromosomes (Table 1, Figure 1) with length from 2.44 Mb to 9.73 Mb. The BUSCO evaluation showed that 97.6% of Arthropoda genes was identified completely in the *D. dubium* assembly (Table 1). The content of repetitive elements is ∼10.37%, lower than the 14.73% and 22.10% in *D. magna* and *D. pulex* respectively, but higher than the 6.27% in *D. celebensis* (Table S4). In total, 15,465 coding genes were predicted in *D. dubium*, similar with the gene number of *D. celebensis* (15,427 genes),^8^ but less than that of *D. pulex* (30,907 genes).^4^ 97% of BUSCO Arthropoda genes were completely contained in the geneset (Table 1), indicating a high quality of our prediction. The difference genes with *D. pulex* should be mainly non-coding genes.

**Table 1:** Summary of *D. dubium* genome assembly

**Genome assembly statistics**	

Assembly length	101,770,839 bp
Number of contigs	226
Contigs N50	1,304,868 bp
Longest contig	5,615,238 bp
Number of scaffolds	55
Scaffolds N50	4,319,657 bp
Longest scaffold	9,728,177 bp
Top 22 scaffolds length	98,923,360 bp
BUSCO complete percentage for genome	97.6%

**Genome characteristics**	

GC content	45.7%
Content of repetitive elements	10.4%
Predicted protein-coding gene number	15,465
BUSCO complete percentage for gene set	97.0%

### Genome evolution

**Figure 2:**
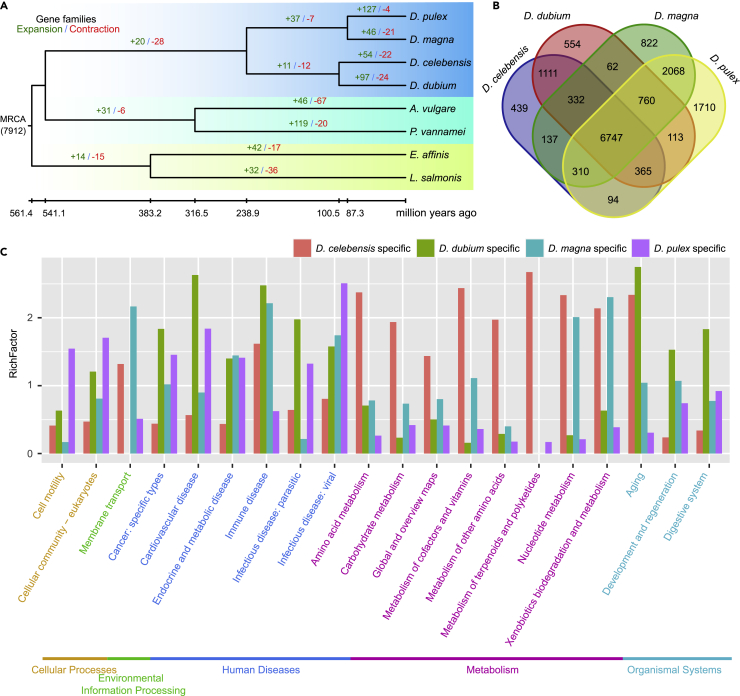
Evolution landscapes of *D. dubium* (A) Phylogenetic tree, estimated divergence time, and estimated number of gene families with significant expansion/contraction evolution (CAFE, viterbi p<=0.05). (B) Venn map of shared and specific gene families among four water fleas. (C) KEGG function enrichment at level 2 category of specific genes in *D. dubium*, *D. celebensis*, *D. magna*, and *D. pulex* respectively. The path name is colored according to the level 1 category. The “RichFactor” = (number of specific genes in this pathway/number of all annotated specific genes)/(number of geneset genes in this pathway/number of all annotated genes of the whole geneset).

To explore the diversification of Cladocera, we compared the *D. dubium* with seven published genomes, including one species of *Diaphanosome* (*D. celebensis*^8^), two of *Daphnia* (*D. pulex*^4^ and *D. magna*^27^), two of Malacostraca (*Penaeus vannamei*^28^ and *Armadillidium vulgare*^29^), and two of Copepoda (*Eurytemora affinis*^30^ and *Lepeophtheirus salmonis*^31^). We identified 1,294 one-to-one orthologous genes and constructed phylogeny trees for these species. The phylogenetic analysis showed that *D. dubium* is closer to *D. celebensis*, and the following sister group is *Daphnia,* as expected.^32^ The two *Diaphanosoma* species was estimated to diverge at Cretaceous period (∼101 million years ago), and *Diaphanosoma* and *Daphnia* was estimated to diverge at Triassic period (∼239 million years ago, Figure 2A). Gene clustering analysis showed lesser orphan genes in *D. dubium* (1,900) than in *D. pulex* (7,743), whereas a similar number of universal orthologous genes (existing in all 8 species) in *D. dubium* (3,916, Table S8) and in *D. pulex* (4,102), implying the gene number difference between two gene sets should be because of non-coding genes or un-conserved genes. Gene variations in sequence and/or copy number are important materials related with adaptive evolution. Here, 554, 439, 822, and 1,710 species-specific gene clusters were identified in *D. dubium*, *D. celebensis*, *D. magna*, and *D. pulex*, respectively (Figure 2B). Compared to the three water fleas, *D. dubium* had a higher ratio of lineage-specific genes involved in the digestive system (Figure 2C). This suggests that food preference may be one major difference between *D. dubium* and other water fleas. The top enriched KEGG pathway of *D. dubium-*specific genes is apoptosis pathway (hypergeometric test, adjusted p-value = 9.13E-15, Table S9). This is an important mechanism to eliminate cells damaged by metabolic or environmental stresses, indicating the response to some environmental stresses may be different in *D. dubium*. 97, 54, 46, and 127 gene clusters that expanded significantly (Viterbi p-value < 0.05) were detected in *D. dubium*, *D. celebensis, D. magna*, and *D. pulex*, respectively. For the expanded genes in *D. dubium*, the most enriched KEGG class is “xenobiotics biodegradation and metabolism” (Figure S1), which is an important process to metabolize xenobiotics, such as the degradation and effluxion of many toxic substances. In summary, these specific and expanded genes provide attractive clues to explore the adaptive biological processes and the underlying molecular mechanisms in *D. dubium.*

### Expansion of EC-SOD genes benefit dispersal of *D. dubium*

**Figure 3:**
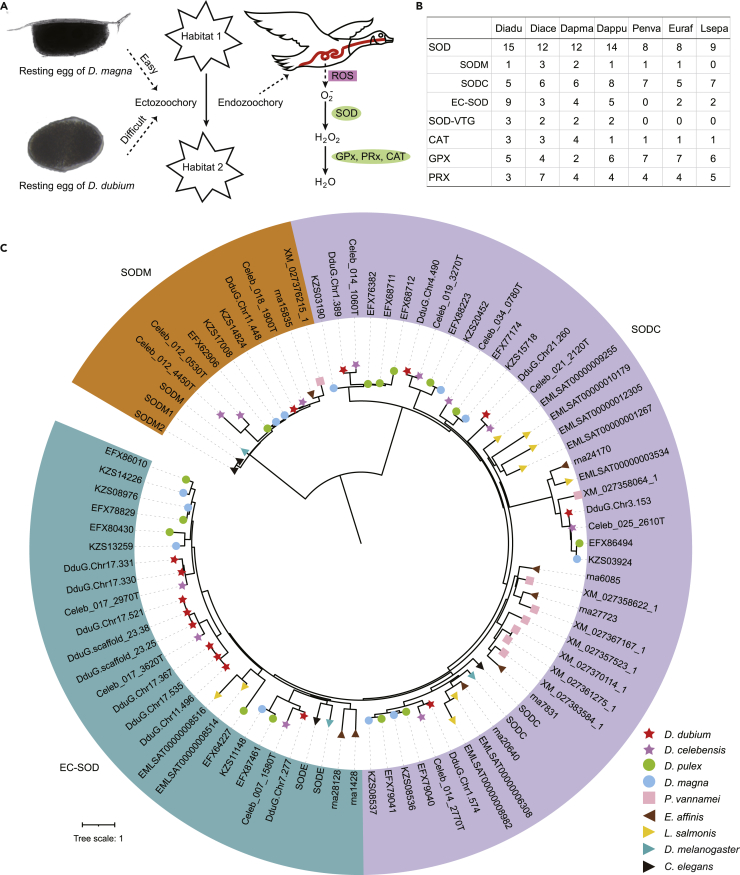
Annotation of anti-ROS genes and phylogenomic analysis of *SOD* genes (A) A photo of resting egg of *D. dubium* and *D. magna* respectively and schematic overview of the anti-ROS pathway. (B) The gene number of four mainly anti-ROS enzymes in seven species. Abbreviation: Diadu, *D. dubium*; Diace, *D. celebensis*; Dapma, *D. magn*a; Dappu, *D. pulex*; Penva, *P. vannamei*; Euraf, *E. affinis*; Lsepa, *L. salmonis*. (C) The phylogeny tree of *SOD* family. The colored geometric shapes at the end-point of branches represent species.

Its broad geographic distribution and disconnected habitats separated by sea or terrestrial barriers indicate the existence of bird-mediated dispersal in *D. dubium*. But unlike species of *Daphnia* with barb-hooked resting eggs (Figure 3A), *D. dubium* has smooth propagules (Figure 3A), implying that dispersal of its resting egg through adhering to waterbirds (ectozoochory, external transport) may be difficult. On the other hand, it has been reported that endozoochory (internal transport) by waterbirds is a viable alternative for many invertebrates,^16^^,^^17^ and the resting eggs of *Daphnia galeata mendotae* and *D. pulex* could hatch after passage through the digestive tract of some fishes, fish-eating birds, and amphibious animals.^33^ Therefore, it could be expected that endozoochory is available to *D. dubium*, and it may be more important for *D. dubium* than for *Daphnia*. However, endozoochory means the resting eggs will face the complex and severe stresses from the gut tract whose function is nutrient absorption and the first line of defense against ingested pathogens. Reactive oxygen species (ROS) or molecules, such as oxygen radicals and superoxide, is constitutively produced in the gut and is an important innate defense system for aerobic organisms.^34^^,^^35^^,^^36^ In addition, ROS could be generated endogenously in all aerobic organisms, and low concentration of ROS is important signals and beneficial for a series of physiological activities.^37^ While adverse internal and external stresses, such as cryopreservation,^38^ radiation,^39^ and toxins,^40^^,^^41^ always cause an unbalanced high level of ROS and damage to organisms. Both the potential high intracellular ROS stress induced by unfitness circumstance of gut (such as acidic condition, digestive enzymes attack) and the intrinsic high extracorporeal ROS stress from the gut should be fatal challenge for resting eggs in the process of internal transport.

The typical ROS is superoxide radical, could be converted into non-reactive water through two steps: into hydrogen peroxide by superoxide dismutase (SOD) firstly, and then into water by glutathione peroxidase (GPX), catalase (CAT), or peroxiredoxin (PRX) (Figure 3A). Compared with the three other water fleas, *D. dubium* had the highest number of SOD domain containing genes (Figure 3B). Of interest, the vitellogenin (Vtg), is the precursor of vitellin which is the major group of yolk proteins and plays a key role in embryonic development and reproduction for oviparous animals.^42^ We found a SOD domain incorporated into Vtg in water fleas^43^^,^^44^ and another *Branchiopoda* species, *Artemia parthenogenetica*.^45^ Here, 3 fusion genes of *SOD* and *Vtg* (*SOD-VTG*) were identified in *D. dubium*, more than the 2 members in the three other water fleas (Figure 3B). The *Vtg*s of the four non-cladoceran species do not contain *SOD*, implying that the *SOD* fusion event happened in the ancestor of *Branchiopoda*. According to the localization, SOD could be classified into three isoenzymes – mitochondrial SOD (SODM, also named SOD2), cytosolic SOD (SODC, also named SOD1), and extracellular SOD (EC-SOD, also named SOD3). *EC-SOD*s contribute the major number difference of this family. There were 9, 3, 4, and 5 *EC-SOD*s in *D. dubium*, *D. celebensis*, *D. magna*, and *D. pulex*, respectively, and more than the number in the compared species in Malacostraca and Copepoda (Figures 3B, 3C, and S2). The phylogenetic tree showed that two sub-clades contain *EC-SOD*s from all four water fleas, indicating there were already at least two members in the ancestor of water fleas. In addition, some members cluster closest with members from the same species (*D. dubium*) or same genus (*Daphnia*, *Diaphanosome*), indicating these expansion events happened independently.

The SOD containing subunits of *SOD-VTG* was detected in *A. parthenogenetic*’s diapause cysts but not in non-diapause eggs, suggesting that the SOD domain in *SOD-VTG* plays an important role in embryos during the diapause process.^45^ Besides, a comparison of mRNA expression between the resting (dormant) and amictic (non-dormant) eggs of a rotifer, *Brachionus plicatilis*, showed that the up-regulated transcripts in resting eggs had the maximum number in antioxidant catalog, indicating that the antioxidant activity played an important role in the dormant process.^46^ The fusion evolution of *SOD* and *Atg* may be one great ‘innovation’ to improve the stress-bearing capacity for the resting eggs of *Branchiopoda*. In *D. dubium*, one more duplication may improve the synthesis speed of SOD-VTG, and therefore is one positive effort to adapt a relative more acute stresses through reducing the generation time of resting eggs. EC-SOD will be transported to the extracellular milieu immediately when produced, and function as an antioxidant against environmental ROS stresses. Earlier studies suggested that EC-SODs play a key role in defending against host-generated ROS in many parasitic helminths.^47^ In nematodes, *EC-SOD* exhibits the largest polymorphism evolution among these antioxidant enzyme genes, including copy number, sequence identity, and gene structure.^47^ In three species of Strongyloides, *EC-SOD* is the only antioxidant enzyme that shows a higher expression in parasitic stage than in free-living stage.^47^ In clade V nematodes, the gene expansion events of *EC-SOD* happened in many parasitic species, such as hookworms and lungworms. In genus *Angiostrongylus*, *EC-SOD* genes experienced expansion and diverged into two clades that showed specific expression during the invasion stage of the intermediate host (snails) and the definitive host (rat) respectively. Moreover, the expanded *EC-SOD*s in *Angiostrongylus* with expression at the snail-invasion stage, showed convergent evolution at some amino acid sites with *EC-SOD*s from five flukes that also need a snail as intermediate host.^48^ Also, the number of these convergent *EC-SOD*s exhibits a positive association with the spectrum of snails that they can invade. One expression study of three key antioxidant enzymes in a blood fluke found that the *EC-SOD* has the largest differential expression between the resistant and susceptible snail infection.^49^ In summary, these studies spanning nematodes and platyhelminthes point that the EC-SODs act as an important resistant factor for some parasitic helminths. Although water fleas are not parasitic, the endured ROS stress is the same from the digestive tract of carrier animals. Thus, the expansion of *EC-SOD*s in water fleas may be one adaptive factor for their endozoochoretic dispersal. The more drastic expansion may give *D. dubium* a stronger dispersal ability carried by up-species in the food chain, such as tolerating longer time in the digestive tract or enduring more species.

### Adaption to eutrophic environments

Through investigating in 19 subtropic reservoirs (Guangdong Province of China), we previously found that *D. dubium* was disadvantaged or even not detected in oligotrophic reservoirs, whereas it dominated in eutrophic reservoirs.^18^ An investigation in 33 water bodies in highly urbanized areas of Philippine, where most of them were subjected to water quality degradation, *D. dubium* was found to be one of the most frequently observed cladocerans.^19^ In one lake from south of South Korea, the abundance of *D. dubium* increased behind cyanobacterial blooms.^20^ In freshwater, cyanobacteria are the dominant group of phytoplankton in eutrophic waters,^23^ compared to eukaryotic algae in oligotrophic waters. Cyanobacterial blooms are well known because of the threat they pose to livestock and humans because of their toxic metabolites.^24^^,^^50^ Besides, the content of numerous biochemical components is different between cyanobacteria and eukaryotic algae, such as lipids and proteins.^25^ Thus, we focused on detoxification and digestion-related genes to investigate adaptative mechanisms to eutrophic habitats.

**Figure 4:**
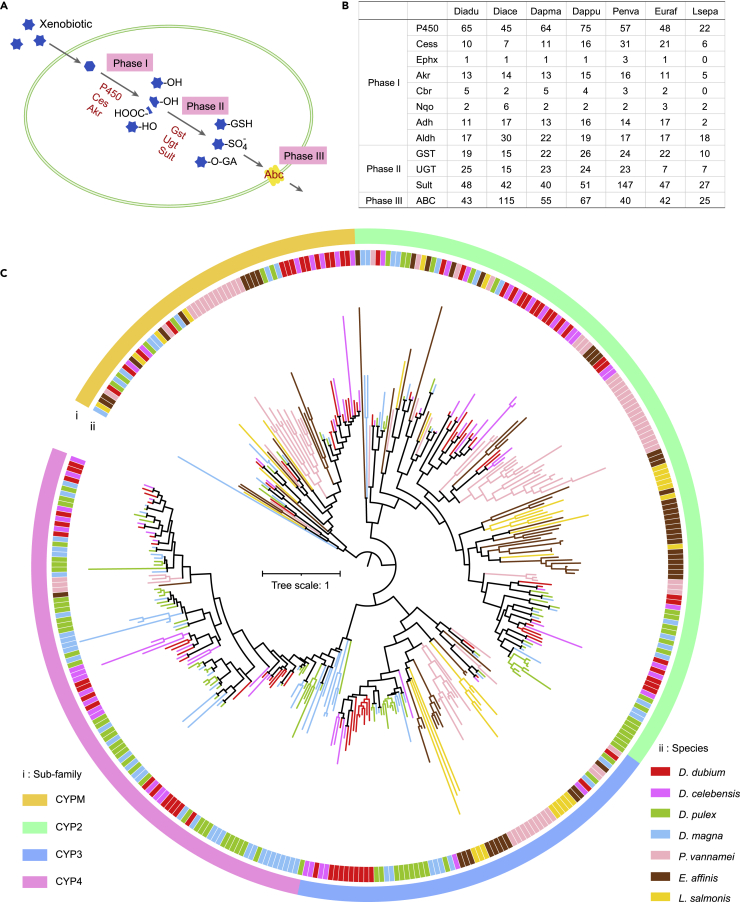
Annotation of twelve main detoxification genes and phylogenomic analysis of *CYP* genes (A) An overview of the xenobiotics biodegradation pathway. (B) The gene number of twelve main xenobiotic-processing genes (XPGs) in seven species. Species abbreviation: Diadu, *D. dubium*; Diace, *D. celebensis*; Dapma, *D. magn*a; Dappu, *D. pulex*; Penva, *P. vannamei*; Euraf, *E. affinis*; Lsepa, *L. salmonis*. Gene abbreviations: Ces, carboxylesterase; Ephx, epoxide hydrolase; Akr, aldo-keto reductase; Cbr, carbonyl reductase; Nqo, NADH:quinone oxidoreductase; Adh, alcohol dehydrogenase; Aldh, aldehyde dehydrogenase; GST, glutathione-S-transferase; UGT, UDP-glucuronosyltransferase; Sult, sulfotransferase; Abc, ATP-binding cassette transporter. (C) The phylogeny tree of *CYP* family. The track “i” represents sub-family classification, the track “ii” represents species.

Metabolic detoxification is a process through which organisms transform foreign substances into metabolites that are less toxic and easier to excrete through detoxification enzymes, and then release the metabolites using transporter proteins.^51^ Overview of xenobiotic biodegradation and metabolization could be summarized in three phases: Introducing a small functional group (such as hydroxy, aldehyde, and carboxyl) to xenobiotic substrates through oxidation, reduction, and hydroxylation; conjugating Phase I modified xenobiotics with bio-subunits (such as glucuronide, sulfate, glutathione, etc.) to increase hydrophilicity; and excreting outside of cell (Figure 4A). Cytochrome P450 (*CYP*) genes play a pivotal role in the metabolization or detoxification of xenobiotics and comprise the largest xenobiotic-processing family.^26^^,^^52^ They continue to receive the maximum attention in terms of the studies of their role involved in drug metabolism in humans which have been estimated to participate in about 75–80% of drug metabolism,^53^ and in insecticides and phytochemicals detoxication in insects.^54^ We identified 65, 45, 64, and 75 *CYP*s in *D. dubium*, *D. celebensis*, *D. magna*, and *D. pulex*, respectively (Figure 4B), showing differences in copy number occurring within both *Diaphanosoma* and *Daphnia*. Furthermore, the phylogenetic analysis showed there are only three clades (two in *CYPM* clan and one in *CYP3* clan) comprising the water fleas except *D. celebensis* and there are multiple species/genus-specific clusters (Figure 4C), indicating that the difference in *CYP* gene number between *D. celebensis* and the other water fleas is not mainly because of the gene loss evolution in *D. celebensis* but because of gene expansion events in other species. Although *D. dubium* and *D. magna* have comparable *CYP* numbers, their evolution processes are quite different. *Daphnia* had drastic expansion in *CYP4* clan, slight expansion in *CYP2* and *CYP3* clans. In comparison, *Diaphanosoma* duplicated in all four clans and evolved more members in *CYP2* and *CYPMito* clans than *Daphnia*. At species level, *D. pulex* evolved additional members in *CYP2* and *CYP3* clans than *D. magna*, and *D. dubium* evolved more members in *CYP3* and *CYPMito* clans than *D. celebensis.*

**Figure 5:**
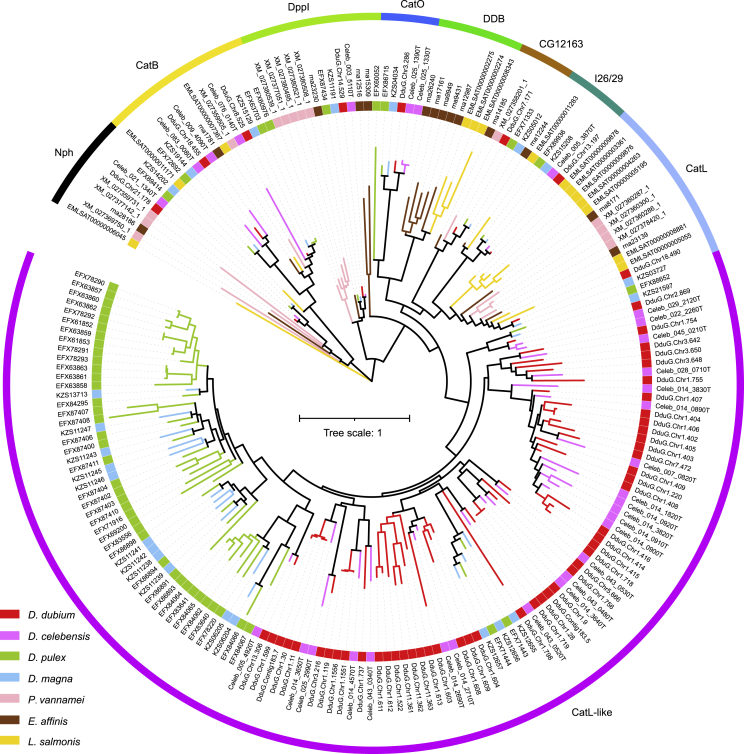
The phylogenomic analysis of cathepsin C genes The colored branches and rectangles at the end-point of branches represent species. The colored outer tracks represent sub-family classification based on the best hit to MEROPS database. Abbreviations: CatB, cathepsin B; CatO, cathepsin O; CatL, cathepsin L; Nph, non peptidase homologues; DppI, dipeptidylpeptidase I; I26/29, insect 26/29 kDa peptidase.

In digestion genes, we identified and compared a set of enzymes that metabolize carbohydrates, lipids, and proteins (Table 2). As the outer barrier of the cell, cell wall is the first barrier for the digestion of algae. Cellulose is a major component for most of the eukaryotic algal cell wall, and accounts for more than 70% in some species.^55^ The process of lignocellulose digestion can be briefly described as follows: β-1,4-endoglucanases (glycoside hydrolase family 5 (GH5), GH9) depolymerize fibrils into individual cellulose polymers, then β-1,4-exoglucanases (GH7) cleave cellulose polymer into 2–4-mer subunits; finally, β-glucosidases (GH5, GH9, or GH30) break cellobiose into glucose units.^56^ Compared to the two species of *Daphnia*, a similar gene number was found for β-1,4-endoglucanases and β-glucosidases in *D. dubium*. However, there is only 1 β-1,4-exoglucanases in *D. dubium*, less than the 3 and 7 in *D. magna* and *D. pulex* respectively (Table 2). It seems likely that *D. dubium* may have a poorer digestive capability for cellulose containing algae than the two species of *Daphnia*. *D. celebensis* has the least (or co-least) gene number for all three enzymes, which is in accord with diatoms that lack cellulose dominate in marine phytoplankton. On the other hand, the major component of cell wall of cyanobacteria is peptidoglycan, similar to Gram negative bacteria. Also, the protein content of whole cell is generally higher in cyanobacteria (could up to >60%) than in eukaryotic algae.^25^ Thus, protein digestion capability should be one important factor for water fleas to survive cyanobacterial blooms. Serine proteases, including trypsins and chymotrypsin, are the dominant digestive enzymes in *Daphnia*^57^ and many crustaceans.^58^ Besides, cysteine proteases and aspartic proteases have also been reported in the gut of organisms as diverse as platyhelminths,^59^ nematodes,^60^^,^^61^ and arthropods.^62^ Here, these three classes of proteases were identified, and *D. dubium* was found to have the maximum member of cysteine proteases (Table 2). Within two species of *Daphnia*, *D. pulex* was found to have more than two-fold number of cysteine proteases than *D. magna*. The phylogeny tree showed the expanded cysteine proteases of *D. dubium* and *D. pulex* were divided into different clades (Figure 5), illustrating that the duplicated events in *D. dubium* and *D. pulex* are independent. All the expanded cysteine proteases in the two water fleas had the best hit to cathepsin L (Figure S3). Similar with *CYP*s, the expanded cysteine protease genes were clustered into more clades in *D. dubium* than in *D. pulex* (2 vs 1, Figure 5).

**Table 2:** The annotation statistics of digestion-related enzymes

	*D. dubium*	*D. celebensis*	*D. magna*	*D. pulex*	*P. vannamei*	*E. affinis*	*L. salmonis*
GH5	6	1	4	5	1	5	1
GH7	1	1	3	7	0	2	0
GH9	5	2	5	5	2	4	0
GH15	2	2	2	2	3	3	2
GH16	7	3	8	10	3	2	0
GH18	26	21	28	26	27	15	7
GH31	10	9	14	9	7	7	5
Serine proteases	152	115	146	244	239	191	166
Cysteine proteases	57	31	23	53	14	14	14
Aspartic proteases	2	1	4	3	3	3	3
Phospholipase	9	9	8	13	14	17	14
Triacylglycerol lipase	20	15	20	21	15	6	21

*D. pulex* and *D. magna* also occurred widely in eutrophic waters.^63^^,^^64^ *D. pulex* appears to tolerate to a more diverse cyanobacterial species than *D. magna,* confirmed by a culture comparison of feeding six different cyanobacteria genera.^65^ Here, *CYP* genes, the important detoxification genes, were found to expand in both species of *Daphnia*, and evolved more members in *D. pulex*. Generally, different subfamilies or members of *CYP*s usually exhibit differential selectivity in substrate.^52^ Based on the gene number variation of the expanded *CYP*s, the two species of *Daphnia* may have a drastically enhanced metabolic ability for some substrates, and *D. pulex* gets additional enhancement for more substrates. *D. dubium* has a comparative expansion number in four clans, indicating it may have a more balanced biodegradation or detoxification ability for a broader spectrum of xenobiotics. Serine proteases are the largest digestive protease family in water fleas, as well as in the compared Malacostraca and Copepoda (Table 2). Whereas plenty of cyanobacterial species produce protease inhibitors, among which the majorities are serine protease inhibitors with negative effects on the growth of *Daphnia*.^66^^,^^67^ It has been reported that cathepsin L-like proteinases (belonging to cysteine proteases) are the dominant digestive proteases in the brown shrimp (*Crangon crangon*), which shows good adaption in a highly variable environment.^58^^,^^68^ We suggest that *D. dubium* and *D. pulex* could have evolved to a stronger compensating capacity to the digestive system of serine proteases through the expansion of cathepsin L-like genes, which largely improves the efficiency in digesting cyanobacteria and other food sources. In summary, the potential higher detoxification and protein digestion ability may enhance their tolerance to eutrophic waters. On the other hand, these expansion events are mainly independent and the evolution pattern is different between *D. dubium* and *Daphnia*, providing one new example of convergent evolution.

### Expansion of *p53*-like and spermine synthase genes

Except for individual survival, successfully breeding a next generation is another precondition to adapt to varied or new habitats. The broad geographic distribution carries multiple environmental stresses and challenges the reproduction of *D. dubium*. In the genome of *D. dubium*, two expanded genes which are associated with reproductive evolution were found.

**Figure 6:**
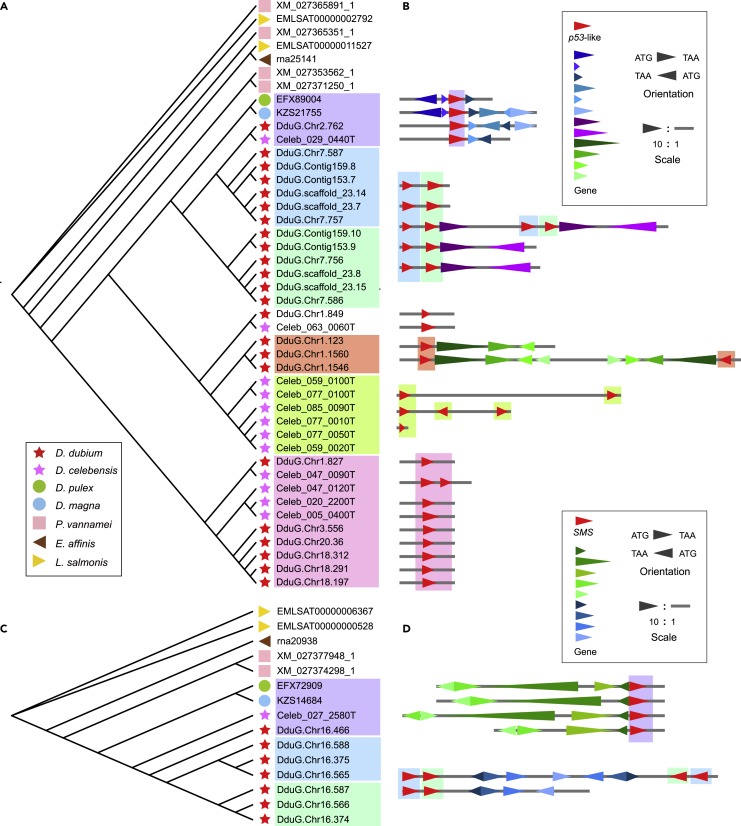
The evolution of *p53*-like and *SMS* genes (A) The phylogeny tree of *p53*-likegenes. (B) Genome syntenic blocks of *p53*-like genes in water fleas. Triangles represent genes and are distinguished by color based on gene name. The red triangles represent *p53*-like genes which are at the same horizon line on the left phylogeny tree except the multiple *p53*-like genes shown in one block. The background color under *p53*-like genes is accordance with the background color in left phylogeny tree. The base to apex angle of triangle represents encoding direction. Five genes on upstream and downstream of *p53*-like are checked and the un-reappeared genes are not shown. Proportional scale, exon region: non-exon region = 10: 1. (C) The phylogeny tree of *SMS* gene. (D) Genome syntenic blocks of *SMS* genes in water fleas. The genes are colored independently with genes in panel B. The red triangles represent *SMS* genes.

The first noticed gene is *p53*-like gene because they are involved in nine out of ten top enriched KEGG pathways of the lineage-specific genes in *D. dubium* (Table S9). The *p53* gene is a tumor suppressor gene which acts as a super stress signals aggregator and integrating responder, resulting in DNA repair or cell apoptosis.^69^^,^^70^ The input signals include extensive internal and external cellular stresses, such as DNA damage, telomere erosion, aging, starvation, hypoxia, radiation, infections, and so on. On the other hand, the *p53* gene becomes oncogene when its function is limited or changed. The mutated *p53* gene could be found in more than 50% of tumors. It has two paralogous genes in vertebrates, *p63* and *p73* genes, whereas most invertebrates have only a single orthologous member, a *p53*-like gene.^69^ Of interest, the *p53*-like gene duplicated repetitively in two species of *Diaphanosoma*, including the common expansions in their ancestor and the independent expansions in each species after their speciation through tandem duplications and dispersive duplications, and finally obtained a total of 23 and 12 members in *D. dubium* and *D. celebensis*, respectively (Figures 6A and 6B). The three members in vertebrates exhibit diverse functions in different tissues, such as *p53* in somatic and *p63* in germ line. However, it was considered with the same purpose, the fidelity, which reduces mistakes of new cells or offspring by regulating homeostasis (unbalanced homeostasis enhance mutations rate), by DNA repair or by cell death.^69^ It has been reported that the *p53*-like gene of invertebrates expresses tissue specifically in the germ line, and has similar functions with *p63* of vertebrates. One example, starvation activates the *p53*-like gene which kills germ cells in flatworms, and the *p63* gene kills germ cells in anorexia human females.^71^ As a key central node in many stress responding networks, the expansion of the *p53*-like gene may be a positive evolution to deal with multiple or varied external stresses in *D. celebensis* and *D. dubium.*

The second highlighted gene is spermine synthase gene (*SMS*) which catalyzes the production of spermine from spermidine. *D. celebensis* and the two species of *Daphnia* have only one *SMS* gene, whereas *D. dubium* got 6 extra copies through tandem and dispersive duplication events (Figures 6C and 6D). Spermine has been found in organisms including animals, plants, fungi, bacteria, and archaea^72^, after it was first found as a component of seminal plasma.^73^ One of natural function of spermine is as a direct free radical scavenger,^74^ which could reduce damage from ROS. In a canine spermatozoa cryopreservation study, additional spermine treatment benefits spermatozoa significantly, decreasing both intracellular and extracellular ROS concentrations, preventing apoptosis and multiple other adverse effects caused by cryopreservation.^75^ Increased *SMS* members should enhance the synthesis ability of spermine in *D. dubium*, and therefore improve resistance to ROS and protect its germ cells.

Taken together, through the expansion of *p53*-like and *SMS* genes, as well as the expansion of *SOD-VTG* and *EC-SOD* gens argued above, *D. dubium* may evolve a more adaptable reproductive capability to handle broad and varied environments, such as fluctuating temperature and eutrophication with cyanobacterial blooms.

### Conclusion

We first reported a high quality and chromosome level genome assembly of *D. dubium*, a keystone planktonic water flea for freshwater ecosystems in Asia. Through comprehensively comparative genomic analyses, we found several important genes experienced expansion events which could partly explain the broad adaptability of *D. dubium*, such as wide distribution and dominance in eutrophic waters. First, the expansion of *p53*-like, *SOD-VTG*, *EC-SOD*, and *SMS* genes may improve the reproductive adaptive ability of *D. dubium*. The *p53*-like gene functions in regulating the fate of germ line cells under stress signals. We speculate it may be a positive factor because of the unsophisticated hypothesis that natural selection has its meaning. The actual regulating pathway is an attractive subject in future studies. The other three expanded genes can enhance resistance against the ROS stresses at both enzyme dependent and enzyme independent levels, which may improve the endozoochory (such as by birds) ability. Second, the potential enhancement of detoxification ability and prokaryotic cell digestion ability may partly contribute to its adaptation to eutrophic waters through the expansion of *CYP* and cysteine protease genes. The genomic resources presented in this study help eliminating the gap of the lacking genomes of freshwater Ctenopoda, and will be the footstone for future omics studies of *Diaphanosoma,* the largest genus of the family Sididae of Cladocera*.*

### Limitations of the study

We assembled a high-quality genome of *D. dubium* and performed comparative genomic analysis. We identified several important genes which could provide insights into potential molecular basis of its broad ecological adaptation. However, further studies, such as more omics studies and functional experiments, are needed to determine the underlying molecular mechanisms.

## STAR★Methods

### Key resources table

**Table:** 

REAGENT or RESOURCE	SOURCE	IDENTIFIER
**Deposited data**		

Pacbio sequencing data	This study	CNGBdb: CNP0002203
Hi-C sequencing data	This study	CNGBdb: CNP0002203
RNA sequencing data	This study	CNGBdb: CNP0002203
Genome assemblydata	This study	CNGBdb: CNP0002203
*Diaphanosoma celebensis*data	Kim et al.^8^	http://rotifer.skku.edu:8080/Dc
*Daphnia pulex*data	Colbourneet al.^4^	NCBI: GCA_000187875.1
*Daphnia magna*data	Orsini et al.^27^	NCBI: GCA_001632505.1
*Penaeus vannamei*data	Yuan et al.^28^	NCBI: GCF_003789085.1
*Armadillidium vulgare*data	Chebbi et al.^29^	NCBI: GCA_004104545.1
*Eurytemora affinis*data	Eyun et al.^30^	NCBI: GCF_000591075.1
*Lepeophtheirus salmonis*data	Skern-Mauritzen et al.^31^	https://metazoa.ensembl.org/Lepeophtheirus_salmonis

**Software and algorithms**		

Falcon (v0.2.2)	Pendleton et al.^76^	https://bitbucket.org/znfinger/na12878_architecture/src/master/
Nextpolish (v1.3.1)	NA	https://github.com/Nextomics/NextPolish
3D-DNA (v180922)	Dudchenko et al.^77^	https://github.com/aidenlab/3d-dna
Bowtie2 (v2.2.5)	Langmead and Salzberg^78^	https://github.com/BenLangmead/bowtie2
HiC-Pro (v2.5.0)	Servant et al.^79^	https://github.com/nservant/HiC-Pro
Jurcer (v1.5)	Durand et al.^80^	https://github.com/aidenlab/juicer
BUSCO (v3)	Seppey et al.^81^	https://busco.ezlab.org/
Repeatscout (v1.0.5)	Price et al.^82^	https://github.com/mmcco/RepeatScout
RepeatMasker (v3.3.0)	NA	http://www.repeatmasker.org/
Augustus (v2.5.5)	Stanke et al.^83^	http://augustus.gobics.de/
FgeneSH	Solovyev et al.^84^	http://www.softberry.com/berry.phtml?topic=fgenesh&group=programs&subgroup=gfind
GlimmerHMM (v3.0.1)	Majoros et al.^85^	https://github.com/kblin/glimmerhmm
GeMoMa (v1.9)	Keilwagen et al.^86^	http://www.jstacs.de/index.php/GeMoMa
Genewise (v2.4.1)	Birney et al.^87^	https://www.ebi.ac.uk/∼birney/wise2/
Hisat2 (v2.1.0)	Kim et al.^88^	https://github.com/DaehwanKimLab/hisat2
Stringtie (v1.0.4)	Pertea et al.^89^	http://ccb.jhu.edu/software/stringtie/
TransDecoder	NA	https://github.com/TransDecoder/TransDecoder
EVidenceModeler (v1.1.1)	Haas et al.^90^	https://github.com/EVidenceModeler/EVidenceModeler
TransposonPSI	NA	http://transposonpsi.sourceforge.net
InterProScan (release 5.3)	Zdobnov and Apweiler^91^	http://www.ebi.ac.uk/interpro/download/InterProScan/
BLAST+ (v2.2.26)	Camacho et al.^92^	https://blast.ncbi.nlm.nih.gov/Blast.cgi?CMD=Web&PAGE_TYPE=BlastDocs&DOC_TYPE=Download
OrthoMCL	Li et al.^93^	https://github.com/stajichlab/OrthoMCL
MUSCLE (v3.8.31)	Edgar^94^	http://www.drive5.com/muscle/
trimAl (v1.2)	Capella-Gutiérrez et al.^95^	http://trimal.cgenomics.org/
RAxML (v8.2)	Stamatakis^96^	https://github.com/stamatak/standard-RAxML
PAML (v4.5)	Yang^97^	http://abacus.gene.ucl.ac.uk/software/paml.html
CAFE (v2.1)	De Bie et al.^98^	https://github.com/hahnlab/CAFE
IQ-TREE (v1.6.6)	Nguyen et al.^99^	http://www.iqtree.org/
iTOL	NA	https://itol.embl.de/

### Resource availability

#### Lead contact

Further information and requests should be directed to the lead contact, Bo-Ping Han (tbphan@126.com, tbphan@jnu.edu.cn).

#### Materials availability

This study did not generate new unique reagents.

### Experimental model and subject details

#### Animals

Zooplankton was collected from Liuxihe Reservoir (23°45′56″N, 113°46′53E), a large, semi-oligotrophic reservoir in Guangzhou, China, where the species are challenged with adverse environmental conditions, e.g., high density of fishes, eutrophication, fluctuated temperature and hydrodynamics. Monoclonal of *D. dubium* was isolated and mass-cultured at room temperature.

### Method details

#### Genome sequencing

Thousands of individuals were immediately frozen in liquid nitrogen and stored at −80°C for genome sequencing. Genomic DNA for long reads sequencing was extracted from about 3,000 individuals*.* A library with 20-kb DNA inserts was constructed following the protocol of the PacBio template preparation kit and sequenced using a Pacific Biosciences Sequel instrument. We sequenced using PacBio Sequel platform and obtained 17.52 Gb sequence data with a mean subread length of 10.59 kb (Table S1). High-throughput Chromosome conformation capture (Hi-C) libraries were also created from about 3,000 individuals as described previously.^100^ In brief, cells were fixed with 2% formaldehyde. The cross-linked DNA was digested with MboI and the sticky ends were biotinylated by incubating with biotin-14-dATP and Klenow enzyme. After DNA purification and removal of biotin from unligated ends, Hi-C products were enriched and physically sheared to fragment sizes of 300–400 bp. The biotin tagged Hi-C DNA was pulled down and processed into paired-end sequencing libraries, which were sequenced on the MGI2000 platform with PE150 sequencing. We got a total of 22.4 Gb high quality Hi-C data.

#### Genome assembly

The assembling began from Pacbio reads assembled to Contig by Falcon^76^ (v0.2.2, with parameters: pa_DBsplit_option-x2000-s600; pa_HPCdaligner_option-v-D200-M24-h600-e.75-l4500-s1000-k18). Owing to the high error rate of Pacbio reads, we corrected the raw assembly using Pacbio reads using the Nextpolish software (v1.3.1, https://github.com/Nextomics/NextPolish). The pseudochromosome organization was constructed using Juicer+3d_dna pipeline^77^ with slight modifications. Briefly, Hi-C data were aligned firstly on the *D. dubium* assembly using Bowtie2^78^ (v2.2.5) software. We filtered the unmapped reads, multiple mapped reads, and singleton pairs with only one read mapped to genome for paired reads. The unique paired alignments were transmitted into HiC-Pro^79^ (v2.5.0) software to identify the valid interaction pairs. Besides, we also removed duplicated read pairs. At last, Juicer^80^ and 3d_dna^77^ were used for assembly clustering, ordering, orienting, evaluating, and manual corrections. The completeness of assembly was assessed using Benchmarking Universal Single-Copy Orthologs (BUSCO) pipeline^81^ with orthologs database of arthropoda_odb9.

#### Repetitive elements prediction

We predicted the repetitive elements (REs) using a “Repeatscout + RepeatMasker” pipeline. Firstly, Repeatscout^82^ (v1.0.5) were employed to build a *de novo* consensus repeat library. Secondly, putative protein-coding genes were removed from the library by alignment to the Swiss-Prot database. Thirdly, RepeatMasker (http://www.repeatmasker.org/, v3.3.0) was used to find repeat in the genome against the *de novo* library. We calculated the multiple substitution using the Jukes-Cantor distance JC = −3/4∗log(1–4∗d/3), where d was the divergence estimated by RepeatMasker. We also predicted for 3 published water flea genomes using the same pipeline to reduce system errors in comparison.

#### Gene prediction

Evidence from *de novo*, homolog-based, and RNA-seq predictions were employed to predict protein-coding gene models. a) *De novo* prediction: Augustus (v2.5.5),^83^ FgeneSH,^84^ and GlimmerHMM (v3.0.1)^85^ were employed to predict *de novo* gene models in genome of *D. dubium* respectively. b) Homolog-based prediction: Protein sequences of 3 published species (*D. magna, D. pulex, and L. salmonis*) were downloaded firstly. Then GeMoMa (v1.9)^86^ and Genewise (v2.4.1)^87^ (v2.4.1) were employed to align onto protein sequences and predict gene models respectively. c) RNA sequencing and prediction: We extracted RNA from about 1,500 individuals and constructed a cDNA library using the Illumina TruSeq RNA sample preparation kit according to the manufacturer’s instructions. The RNA sequencing was performed by Illumina Xten platform with PE150 strategy. For the prediction, the RNA-seq reads were aligned to the assembly using Hisat2^88^ (v2.1.0) with default parameters. The transcripts were then assembled using Stringtie^89^ (v1.0.4) and open reading frames (ORFs) were predicted using TransDecoder (https://github.com/TransDecoder/TransDecoder). Finally, the multiple prediction from above three strategies were integrated using EVidenceModeler (v1.1.1)^90^ (EVM) software. We also filtered the transposable elements using TransposonPSI (http://transposonpsi.sourceforge.net) software. The completeness of the geneset was also assessed using BUSCO pipeline.

We annotated the function of predicted genes by aligning to the Swiss-Prot^101^ (release Jun 2019), NCBI Nr (release Sep 2017), and KEGG^102^ (release 89) databases. The gene symbols and pathways were assigned based on the best blast hit against the Swiss-Prot and KEGG databases respectively. GO terms, motifs and domains of protein sequences were annotated using InterProScan^91^ (release 5.3) by searching against publicly available databases, including Pfam, PRINTS, PANTHER, PROSITE, ProDom, and SMART.

#### Gene clustering

Orthologous and paralogous gene clusters were identified using Blastp + OrthoMCL^93^ pipeline. Protein sequences of *Diaphanosoma celebensis*, *Daphnia pulex*, *Daphnia magna*, *Penaeus vannamei*, *Armadillidium vulgare*, *Eurytemora affinis*, and *Lepeophtheirus salmonis* was obtained from public database (shown in key resources table). If a gene had more than one transcript, the longest transcript was used. After the data processing, BLASTp (blast-2.2.26)^92^ was employed to do an all_vs_all alignment for protein sequences of above species and *D. dubium* to identify the potential homologous sequences with E-value < 1e-5. The blast results were clustered into gene clusters using OrthoMCL with default parameters. Based on this cluster result, we identified lineage specific genes in species or clade. We carried out KEGG function enrichment for lineage specific genes through a hypergeometric test using PHYPER package in R (v3.5.1) and did a false discovery rate (FDR) test using QVALUE package in R.

#### Phylogenetic analysis and divergent time estimation

We obtained 1,294 one-to-one orthologous genes among 8 species from the above OrthoMCL cluster result. The protein sequences of each orthologs were aligned using MUSCLE^94^ (v3.8.31) with default parameters. Poorly aligned regions were removed using trimAl^95^ (v1.2) with the parameter “-gt 0.8-st 0.01”. Then, we converted into nucleotide alignment by tracing the coding relationship. We connected all alignments of single orthologs to form a concatenated alignment. Finally, we used RAxML^96^ (v8.2) to construct phylogeny tree under GTRGAMMA model.

We estimated divergence times using MCMCTREE package from PAML software^97^ (v4.5). The Markov chain Monte Carlo (MCMC) process was run for 10,000 iterations with a sample frequency of 5,000 after a burn-in of 5,000,000 iterations, and other parameters were set as defaults. Three calibrating points were applied as follows: (i) Cladocera: 176–326 mya, based on a fossil of Smirnovidaphnia;^103^ (ii) the divergence time between *E. affinis* and *D. pulex* is 472–575 MYA (TimeTree, http://www.timetree.org/); (iii) the divergence time between *D. pulex* and *Armadillidium vulgare* is 526.3–577.5 MYA (TimeTree).

#### Gene expansion and contraction

We identified the expansion and contraction of genes using CAFE (Computational Analysis of gene Family Evolution, version 2.1)^98^ which infers the dynamics of gene family under a stochastic birth and death model. The input files include the table containing gene number of each cluster and species tree with divergence time from above OrthoMCL pipeline and MCMCTREE software respectively. The executed parameter of CAFE used “-p 0.05-t 4-r 10000-filter”. As a result, we selected the gene clusters with size significantly changed for each species and branches (Viterbi p<=0.05).

#### Gene family evolution

The gene cluster and gene expansion analyses produced by the OrthoMCL and CAFE pipeline revealed that some candidate genes experienced copy number variations or the sequence divergent evolution separated from the background species. To further investigate the evolution of candidate genes, we first identified the whole gene families based on motif annotation or Swiss-Prot annotation. Then we constructed a phylogenetic tree for each gene family. We aligned the protein sequences of each gene family using MSUCLE, and filtered the alignments using trimAl with the parameter “-gt 0.8.” The phylogenetic tree was constructed using IQ-TREE^99^ (v1.6.6) software with the default parameters. Finally, we constructed the phylogenetic trees for gene families of *SOD* (Pfam motif: PF00080 and PF00081), *CYP* (Pfam motif: PF00067), cysteine proteases (Pfam motif: PF00112), *p53-*like (Pfam motif: PF00870), *SMS* (InterPro motif: IPR015576). Due to the poor quality of *A. vulgare*’s geneset (72% of completed BUSCO Arthropoda genes), we discarded *A. vulgare*’s genes in phylogenetic analyses of these families. For *SOD* domain containing genes, we filtered copper chaperone for superoxide dismutase genes and *SOD-VTG* genes. It has been reported that the *SOD* domain in *Branchiopoda SOD-VTG* is close to viral and bacterial Cu/Zn *SODs* and thus should be acquired by horizontal gene transfer.^45^ The phylogeny trees were visualized using iTOL (https://itol.embl.de/).

### Quantification and statistical analysis

Quantification and statistical analysis used in the genome sequencing and assembly, genome quality assessment, evolutionary analysis and comparative genome analysis can be found in the relevant sections of the method details.

## Data Availability

The sequencing data and genome assembly of *D. dubium* are deposited into China National GeneBank DataBase with accession number CNP0002203 (CNGBdb: CNP0002203, https://db.cngb.org/search/project/CNP0002203/). Custom scripts and any other relevant data are available from the corresponding authors upon request.
