# Supplementary material for: High-quality genome of Diaphanosoma dubium provides insights into molecular basis of its broad ecological adaptation
Source: iScience. 2023 Jan 18;26(2):106006. doi: 10.1016/j.isci.2023.106006 (PMC9926121; doi:10.1016/j.isci.2023.106006)
Supplement: Document S1. Figures S1–S3 and Tables S1–S10 [file mmc1.pdf]

iScience, Volume *26*

Supplemental information

High-quality genome of *Diaphanosoma dubium* provides insights into molecular basis of its broad ecological adaptation

Meng Xu, Ping Liu, Qi Huang, Shaolin Xu, Henri J. Dumont, and Bo-Ping Han

Supplemental Information

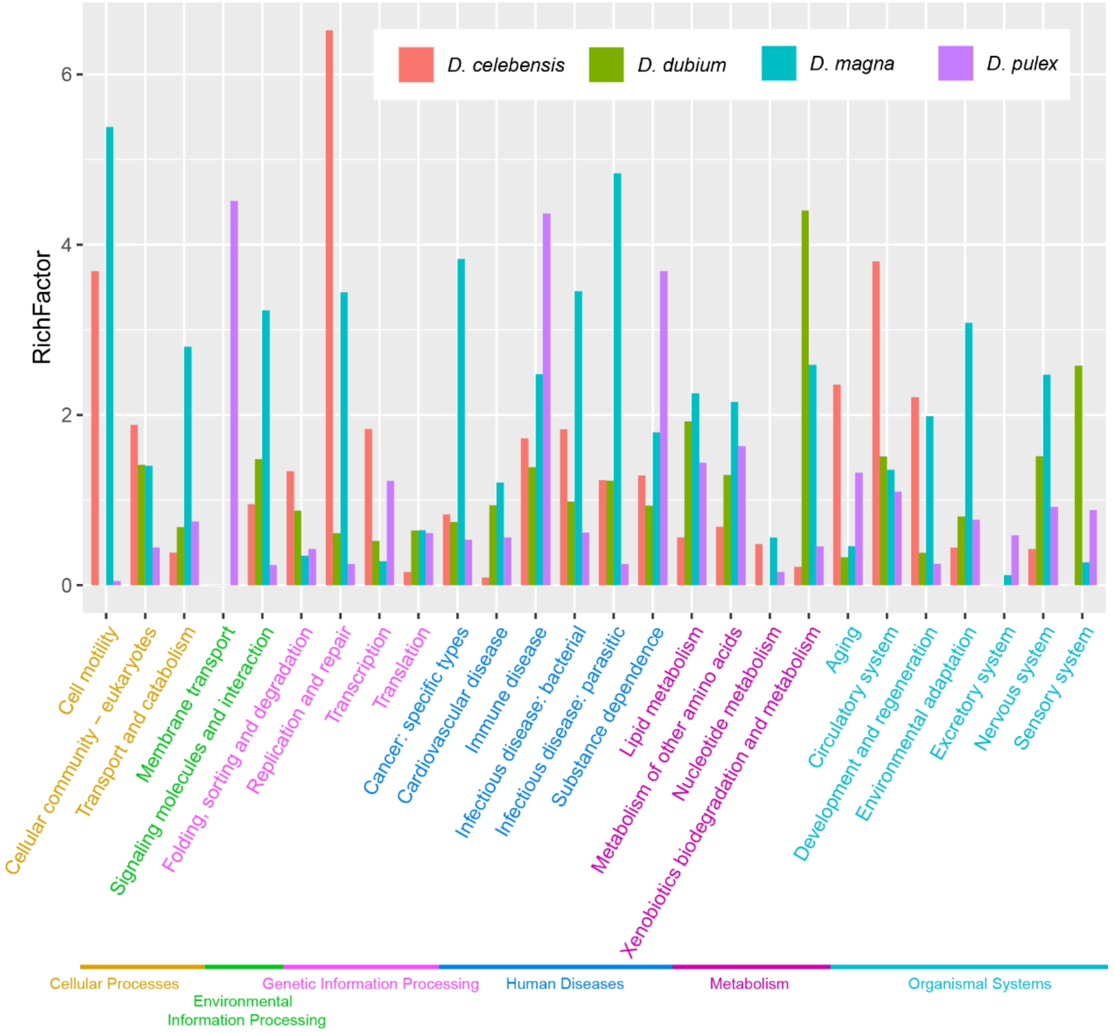

Fig. S1 KEGG function enrichment at level 2 category of significantly expanded (CAFE, viterbi p<=0.05) genes in *D. dubium*, *D. celebensis*, *D. magna*, and *D. pulex*, respectively. The path name is colored according to the level 1 category. The "RichFactor" = (number of expanded genes in this pathway / number of all annotated expanded genes) / (number of geneset genes in this pathway / number of all annotated genes of the whole geneset). Considering visible reason, we only show the KEGG categories with maximum fold difference of RichFactor larger than three. Related to Figure 1.

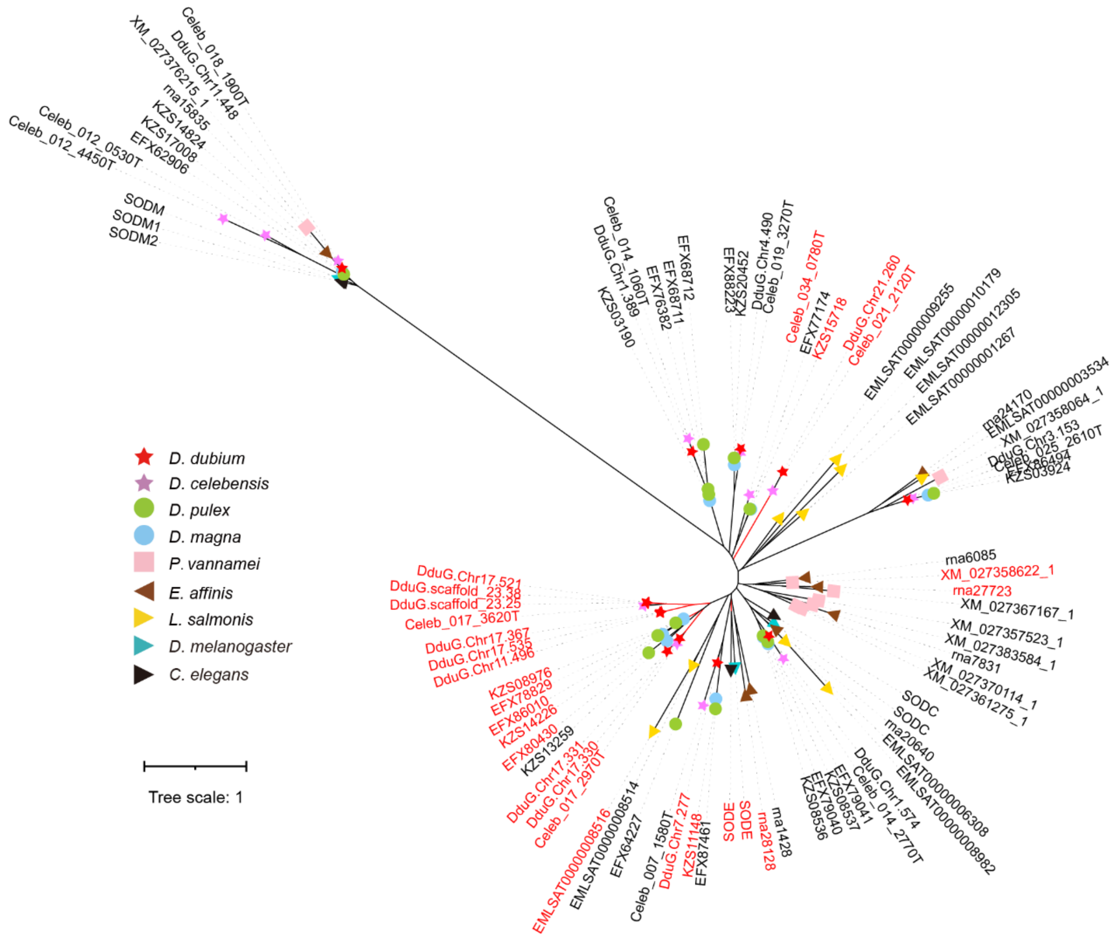

Fig. S2 Unrooted phylogeny tree of *SOD* genes for nine species in Cladocerans, Malacostraca, Copepoda, Insecta, and Nematode. The highlight genes with red color have signal peptide predicted by SignalP (https://services.healthtech.dtu.dk/service.php?SignalP-5.0). Related to Figure 3.

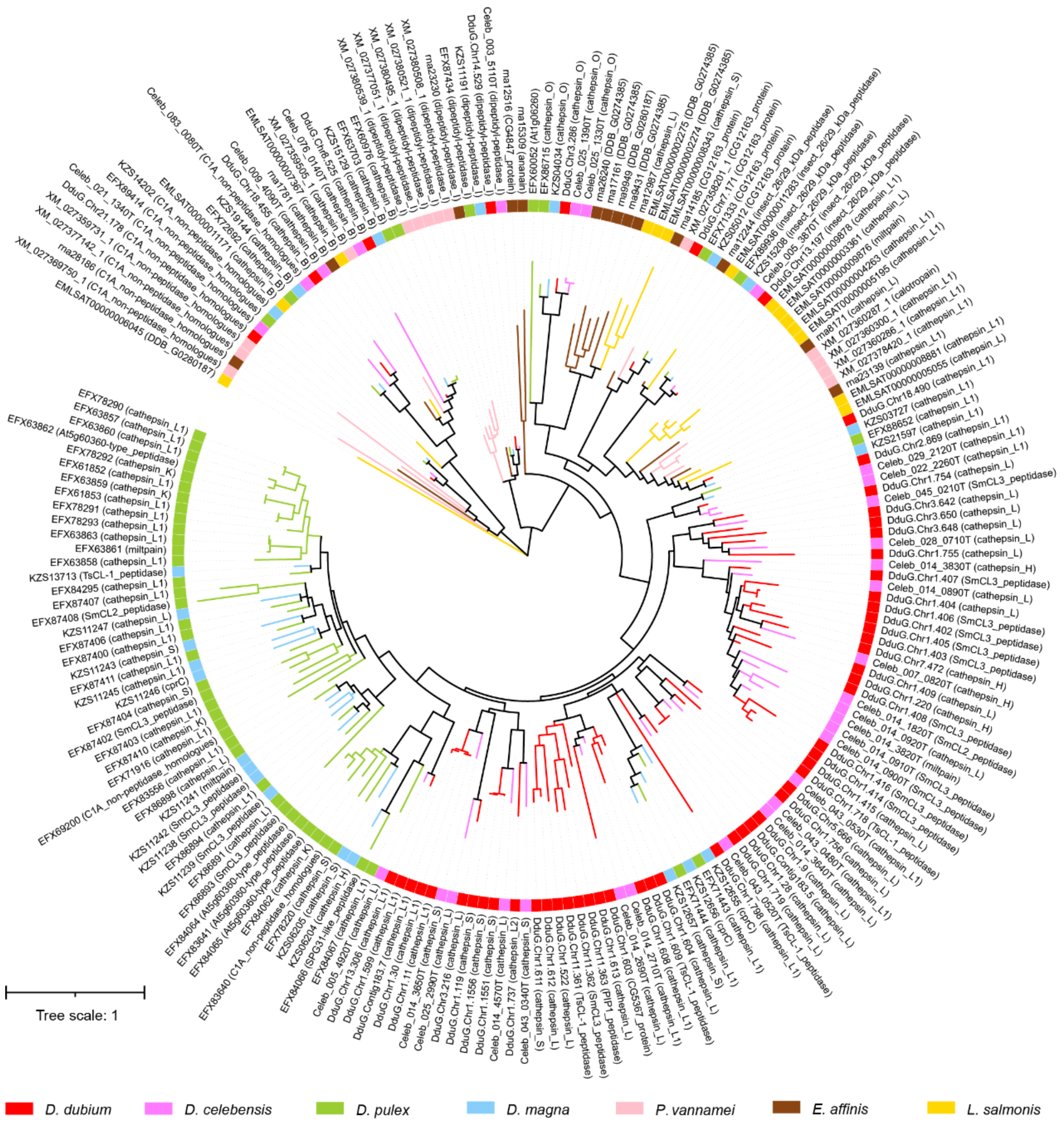

Fig. S3 The phylogeny tree with annotated label of cysteine protease genes in eight species. The best aligned MEROPS proteases are shown in brackets behind of the gene ID. Related to Figure 5.

Table S1 Statistics of Hi-C and PacBio sequencing data for *D. dubium*. Related to Table 1.

Platform	Total bases (Gb)	Total reads	Read length (bp)		
			Max length	Average length	N50 length
Hi-C	22.40	149,306,146	150	150	150
Pacbio	17.52	1,653,406	95,851	10,593	13,250

Table S2 Statistics of genome assembly for *D. dubium*. Related to Table 1.

	scaffold		contig	
	length(bp)	number	length(bp)	number
max_length	9,728,177		5,615,238	
N10	6,242,773	2	3,717,906	3
N20	6,142,465	3	3,375,435	5
N30	5,538,763	5	2,437,993	9
N40	5,240,602	7	1,687,218	14
N50	4,319,657	9	1,304,868	21
N60	4,244,033	11	1,107,508	29
N70	4,119,937	14	859,208	39
N80	3,403,024	17	484,002	56
N90	3,016,504	20	163,504	94
Total_length	101,856,339		101,770,839	
number>2000bp		55		226

Table S3 BUSCO assessing of genome assembly for *D. dubium*. Related to Table 1.

Type	Number	Percent (%)
Complete BUSCOs (C)	1,040	97.6
Complete and single-copy BUSCOs (S)	972	91.2
Complete and duplicated BUSCOs (D)	68	6.4
Fragmented BUSCOs (F)	12	1.1
Missing BUSCOs (M)	14	1.3
Total BUSCO groups searched	1,066	

Table S4 Repetitive elements statistics in genomes of four water fleas. Related to Table 1.

Type	*D. dubium*		*D. celebensis*		*D. magna*		*D. pulex*	
	Length (Bp)	% in genome	Length (Bp)	% in genome	Length (Bp)	% in genome	Length (Bp)	% in genome
DNA	944,717	0.93	586,345	0.59	1,867,809	1.44	4,586,057	2.33
LINE	749,744	0.74	337,105	0.34	844,660	0.65	2,972,306	1.51
SINE	147,079	0.14	159,797	0.16	81,417	0.06	764,844	0.39
LTR	2,031,009	2.00	1,385,537	1.38	7,493,414	5.78	14,385,350	7.29
Other	81	0.00	268	0.00	228	0.00	339	0.00
Unknown	7,515,515	7.38	4,490,440	4.49	9,888,647	7.63	22,941,434	11.63
Total	10,553,726	10.37	6,277,435	6.27	19,087,589	14.73	43,584,290	22.10

Supplementary Table S5 Statistical results of gene predictions for *D. dubium*. Related to Table 1.

Method		TN	ATL	ACL	AEN	AEL	AIL
De novo	AUGUSTUS	16,839	4,331.12	1,740.69	7.57	230.07	394.52
	FgeneSH	21,269	2,023.78	1,298.33	4.77	272.05	192.3
	GlimmerHMM	18,150	4,145.94	1,501.96	4.71	318.89	712.66
Homology	GeMoMa	11,280	4,028.12	1,334.62	5.61	237.79	583.93
	GeneWise	13,923	2,201.15	1,199.77	4.41	272.37	294.09
RNA-seq	transdecoder	9,131	4,016.72	1,765.96	7.1	248.56	368.69
Final set	EVM	15,465	3,538.46	1,538.68	6.52	236.05	362.38

Note: TN, total number of predicted genes; ATL, average transcript length (bp); ACL, average coding sequences length (bp); AEN, average exons number per gene; AEL, average exon length (bp); AIL, average intron length (bp).

Table S6 Statistical results of gene function annotation for *D. dubium*. Related to Table 1.

Type		Number	Percent (%)
Annotation	Swissprot	10,938	70.73%
	Kegg	7,560	48.88%
	KOG	9,394	60.74%
	GO	7,066	45.69%
	NR	11,761	76.05%
	InterPro	10,701	69.19%
Total	Annotated	12,381	80.06%
	Gene	15,465	

Table S7 BUSCO assessing of the gene set of *D. dubium*. Related to Table 1.

Type	Number	Percent (%)
Complete BUSCOs (C)	1,034	97.0
Complete and single-copy BUSCOs (S)	965	90.5
Complete and duplicated BUSCOs (D)	69	6.5
Fragmented BUSCOs (F)	12	1.1
Missing BUSCOs (M)	20	1.9
Total BUSCO groups searched	1,066	

Table S8 Gene cluster statistics for eight species in Cladocerans, Malacostraca, and Copepoda. Related to Figure 1.

Species	Genes number	Clustered genes	Unclustered genes	Universal genes	Family number	Unique families	AGPF
A. vulgare	19,051	14,431	4,620	4,304	7,745	860	1.86
D. magna	24,068	16,005	8,063	3,673	11,238	723	1.42
D. pulex	30,141	22,398	7,743	4,102	12,167	1,581	1.84
E. affinis	19,588	13,132	6,456	3,908	8,835	726	1.49
L. salmonis	13,081	9,233	3,848	3,615	7,270	245	1.27
P. vannamei	22,858	17,225	5,633	4,347	9,010	914	1.91
D. dubium	15,465	13,565	1,900	3,916	10,044	490	1.35
D. celebensis	15,427	11,969	3,458	3,549	9,535	328	1.26

Note: AGPF, Average genes per family.

Table S9 Top 20 enriched KEGG pathways for lineage specific genes of *D. dubium*. Related to Figure 1 and Figure 6.

#Pathway	GN	Qvalue	Level 2 label
Apoptosis*	50	9.13E-15	Cell growth and death
Transcriptional misregulation in cancer*	33	5.62E-12	Cancer: overview
Melanoma*	16	3.93E-08	Cancer: specific types
Small cell lung cancer*	23	1.24E-07	Cancer: specific types
Amyotrophic lateral sclerosis (ALS) *	20	1.24E-07	Neurodegenerative disease
Prostate cancer*	19	1.98E-07	Cancer: specific types
Mitophagy - animal*	16	2.76E-07	Transport and catabolism
Longevity regulating pathway*	19	3.27E-07	Aging
Protein digestion and absorption	32	8.51E-07	Digestive system
Bladder cancer*	13	1.18E-06	Cancer: specific types
Fluid shear stress and atherosclerosis*	28	1.18E-06	Cardiovascular disease
Ferroptosis*	13	1.80E-06	Cell growth and death
Thyroid cancer*	13	2.17E-06	Cancer: specific types
Hepatitis C*	19	2.44E-06	Infectious disease: viral
Platinum drug resistance*	18	2.44E-06	Drug resistance: antineoplastic
Wnt signaling pathway*	26	3.14E-06	Signal transduction
p53 signaling pathway*	18	4.10E-06	Cell growth and death
Chronic myeloid leukemia*	15	4.39E-06	Cancer: specific types
Glioma*	16	6.11E-06	Cancer: specific types
Non-small cell lung cancer*	13	6.11E-06	Cancer: specific types

Note: GN, Gene Number; *, pathways containing *p53* genes. Qvalue, the adjusted hypergeometric test p-value.

Table S10 Top 20 enriched KEGG pathways for expanded genes of *D. dubium*.

Related to Figure 1 and Figure 4.

#Pathway	GN	Qvalue	Level 2 label
GnRH signaling pathway	27	3.81E-12	Endocrine system
Drug metabolism - other enzymes	28	5.32E-12	Xenobiotics biodegradation and metabolism
Ascorbate and aldarate metabolism	17	2.51E-11	Carbohydrate metabolism
Porphyrin and chlorophyll metabolism	17	9.77E-10	Metabolism of cofactors and vitamins
Pentose and glucuronate interconversions	17	1.27E-09	Carbohydrate metabolism
Drug metabolism - cytochrome P450	17	1.27E-09	Xenobiotics biodegradation and metabolism
Thiamine metabolism	10	5.38E-09	Metabolism of cofactors and vitamins
Metabolism of xenobiotics by cytochrome P450	17	5.93E-09	Xenobiotics biodegradation and metabolism
Pertussis	20	9.73E-09	Infectious disease: bacterial
TNF signaling pathway	20	5.31E-08	Signal transduction
C-type lectin receptor signaling pathway	20	1.17E-07	Immune system
Retinol metabolism	17	1.17E-07	Metabolism of cofactors and vitamins
Steroid hormone biosynthesis	17	1.17E-07	Lipid metabolism
Parathyroid hormone synthesis, secretion and action	20	1.33E-07	Endocrine system
Folate biosynthesis	14	4.28E-07	Metabolism of cofactors and vitamins
Influenza A	24	6.13E-07	Infectious disease: viral
Chemical carcinogenesis	17	6.51E-07	Cancer: overview
Phospholipase D signaling pathway	19	2.56E-06	Signal transduction
NOD-like receptor signaling pathway	20	1.16E-05	Immune system
Inflammatory mediator regulation of TRP channels	14	8.78E-05	Sensory system

Note: GN, Gene Number. Qvalue, the adjusted hypergeometric test p-value.
